# Hot Deformation Behavior of Q345 Steel and Its Application in Rapid Shear Connection

**DOI:** 10.3390/ma12132186

**Published:** 2019-07-07

**Authors:** Mengwei Wu, Shuangjie Zhang, Shibo Ma, Huajun Yan, Wei Wang, Qiang Li

**Affiliations:** 1 School of Materials Science and Engineering, Hebei University of Science and Technology, Shijiazhuang 050018, Hebei, China; 2 Hebei Key Laboratory of Material Near-Net Forming Technology, Hebei University of Science and Technology, Shijiazhuang 050018, Hebei, China; 3 School of Mechanical Engineering, Yanshan University, Qinhuangdao 066004, Hebei, China

**Keywords:** the thermal deformation behavior, Arrhenius constitutive equation, dynamic recrystallization model, secondary development, shear connection, microstructural evolution

## Abstract

The high-temperature deformation behavior of Q345 steel is detected by a Gleeble-3800 thermal simulator. The Arrhenius constitutive equation for high-temperature flow stress and the dynamic recrystallization model are constructed. With the secondary development technology, customized modifications are made on existing Deform-3D software. The constructed constitutive model and dynamic recrystallization model are embedded into Deform-3D to realize the secondary development of Deform-3D. The grain size and volume percentage distribution of dynamic recrystallization are obtained by simulating the shear connection process at high temperature and high speed. The results show that the constitutive equation and the dynamic recrystallization model constructed in this paper can be used to predict the evolution of the microstructure. The difference between the prediction results and the experimental data is about 3%. The accuracy of Arrhenius constitutive equation, dynamic recrystallization model and the feasibility of software secondary development are verified.

## 1. Introduction

As an important technical method for material connection, the solid metal connection technology of metal sheets becomes a necessary step for materials to be converted into usable parts and structures, which can realize the direct connection of metal sheets [1]. At present, the mature solid-state connection technologies mainly include accumulative roll-bonding (ARB) [2], superplastic forming/diffusion bonding [3], mechanical bonding [4,5], and friction stir welding (FSW) [6,7]. Shear connection is one of the promising solid-state connection technologies because of its innovative technology, good process and low relative cost.

**Figure 1:**
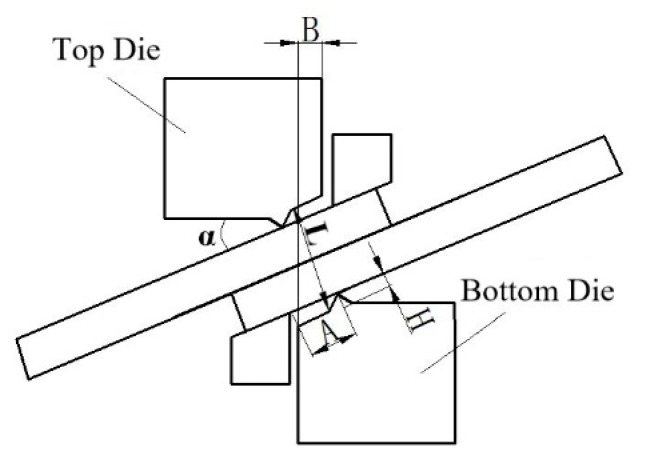
Shear connection diagram.

At present, shear connection technology is mainly applied to the connection of intermediate sheet in endless rolling. A new solid-state connection technology based on the plastic deformation connection process, which was applied to the endless rolling intermediate blank shear connection process, was developed by Posco (Pohang) and Hitachi (Hitachi) Co. Ltd. [8,9,10,11,12]. Relevant papers and reports show that the company mainly studies the connection technology and equipment, and obtains the minimum temperature of effective connection of sheet metal is 980 °C and the thickness of blank is 29–120 mm. The effects of process parameters such as overlap (B), the tilt angle of the sheet (α) and pressure amount (L) and material parameters such as carbon content and oxide content on the interface closure of the joint in Figure 1 are analyzed [12,13,14]. The strength of the connection is proved. Researchers at Hebei University of Science and Technology carried out basic research on the rapid shear connection technology of endless rolling intermediate blanks, and designed the dynamic and static connection mechanism [15,16], which is a relatively simple six-bar and one-slide mechanism, and derived the motion function. Optimization of this mechanism is employed using a genetic algorithm (GA) and the optimization parameters of this mechanism are obtained; the kinematics analysis show that the connection time is less than 0.1 s and the connection thickness is more than 80% of the thickness of the solid-state metal. The change of material structure in the joint zone was analyzed, the dynamic recrystallization is the main driving force for grain refinement is concluded, and the greater the pressure amount, the more intense the dynamic recrystallization and the higher the grain refinement degree. Interfacial bonding is formed by three-stage theory, the increase of the pressure amount, the narrowing of the bonding band, the decrease of voids, the gradual disappearance of the interface and a large number of common grains are formed [17]. The key parameters affecting the rapid shear-extrusion bonding of metals are determined as follows: the overlap (B), the pressure amount (L), the width of the shear edge (A) and the tilt angle of the shear edge (a). Among them, the overlap ranges from 3 mm to 15 mm, the pressure amount ranges from 0.5 to 1.5 times the material thickness, the width of the shear edge from 20 mm to 40 mm, and the tilt angle of the shear edge from 12° to 14° [18,19].

The study of the microstructure evolution of the steel material during hot-working process has great engineering significance. A hardening process caused by plastic deformation and softening process caused by dynamic recovery (DRV) and dynamic recrystallization (DRX) are concomitant in the hot-working process. In particular, dynamic recrystallization behavior has important effects on flow tress and microstructure evolution. It determines the macroscopic mechanical properties of the metal materials [20]. Dynamic recrystallization is an important microstructure evolution behavior during shear connection. Dynamic recrystallized grain size and volume percentage affect the mechanical properties of the connection and its parts. Although many scholars have carried out basic research on shear connection technologies and obtained certain research results, research on the microstructure evolution and dynamic recrystallization process of shear connection has not been reported. Therefore, it is necessary to obtain the grain size and volume percentage of dynamic recrystallization by establishing the constitutive and recrystallization equation of Q345 and the secondary development of Deform-3D. This is of great significance to the optimization of process parameters and the prediction of workpiece performance for the shear connection of endless rolling intermediate blank. This paper aims to obtain the true stress-strain curves of typical carbon steel by the thermal compression test, and construct the Arrhenius constitutive and dynamic recrystallization model. In this paper, the dynamic recrystallization model is put into a user subroutine based on the second development of finite element (FE) software. By the simulation of shear connection process, the dynamic recrystallized grain size and volume percentage at each stage are obtained, and compared with experimental results.

## 2. Arrhenius Constitutive Equation and Dynamic Recrystallization Model

### 2.1. Plastic Deformation Behavior

The experimental material is low alloy steel Q345, which is widely used in industry. Table 1 is the chemical composition of Q345.

The experimental equipment is a Gleeble-3800 thermal simulator developed by DSI Company in the United States (Kansas City, MO, USA), which has the function of thermal dynamic simulation. It can be widely used in the performance analysis of materials in the process of continuous casting, pressure processing, heat treatment, welding and so on. The main parameters are shown in Table 2.

**Figure 2:**
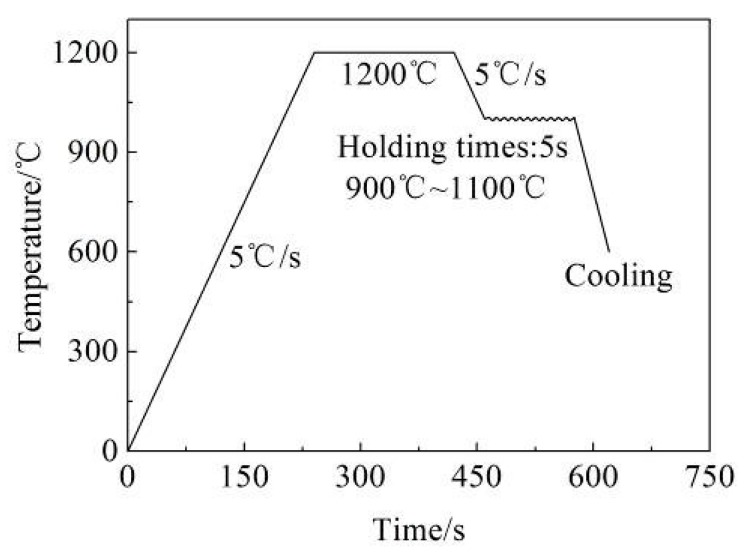
Technological process.

**Figure 3:**
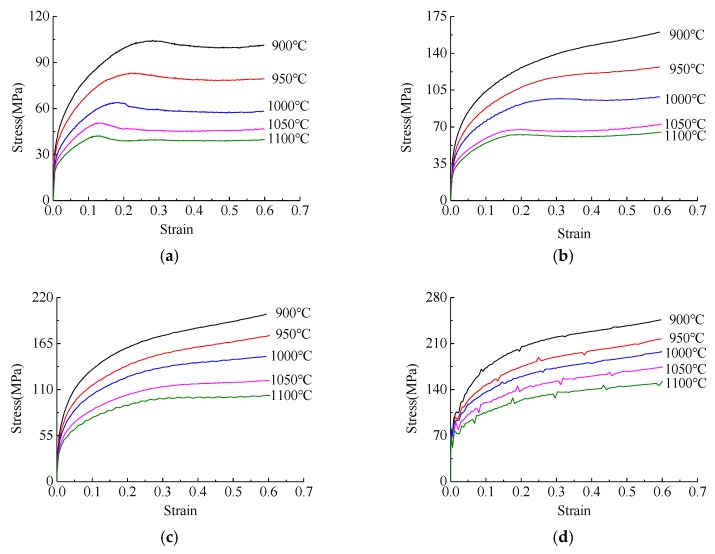
True stress-strain relationship curves of Q345 steel: (**a**) $\dot{\varepsilon }$ = 0.01 s^−1^; (**b**) $\dot{\varepsilon }$ = 0.1 s^−1^; (**c**) $\dot{\varepsilon }$ = 1.0 s^−1^; (**d**) $\dot{\varepsilon }$ = 10 s^−1^.

The specific process flow chart is shown in Figure 2. The compression tests are implemented on a Gleeble-3800 thermal simulation machine at different deformation temperatures of 900, 950, 1000, 1050 and 1100 °C, with strain rates of 0.01, 0.1, 1.0 and 10 s^−1^. The specimen size is 10 mm in diameter and 15 mm in height. Each surface of the specimen is covered with the tantalum foil to minimize friction. The specimens are initially heated to the deformation temperature and then held for 5 min to homogenize the temperature. The true strain reached to 0.6. After the compression test, the compressed specimen is quickly quenched into cold water to keep the microstructure. The quenched specimens are sliced along the axial section, and then polished and etched with a saturation picric for the observation of austenite grain boundaries. Based on the isothermal compression tests, the true stress–strain curves of Q345 steel are plotted, as shown in Figure 3 [21,22]. The deformation behavior of Q345 steel is coincident with the typical rules of low-carbon steel.

From Figure 3, it can be seen that the true stress increases with the increase of true strain at different temperatures at the same strain rate, and the true stress-strain curves have the same trend at different strain rates. At the initial stage of deformation, the true stress value increases sharply with the increase of true strain. With the increase of deformation degree, the increasing trend of flow stress tends to be stable [23]. Under the test condition of strain rate 10 s^−1^, the true stress-strain curves show zigzag changes. The reason for this phenomenon might be that dynamic softening and work hardening alternately lose balance at higher strain rates [24].

### 2.2. Arrhenius Constitutive Equation 

The Arrhenius equation is used to describe the relationship between the corresponding flow stress σ, temperature T and strain rate $\dot{\varepsilon }$ at any strain level of low carbon alloy steel, especially at high temperatures [25,26,27,28,29]. The expression is shown in Equation (1).

(1) $$\dot{\varepsilon }=A_{1}g(\sigma )\exp\left(-\frac{Q}{RT}\right)\;g(\sigma )=\left\{ \begin{array}{l} \sigma^{n_{1}}\\ \exp(\beta \sigma )\\ {\left[\sinh(\alpha \sigma )\right]}^{n} \end{array}\right.\begin{array}{l} \alpha \sigma <0.8\\ \alpha \sigma >1.2\\ \begin{array}{ccc} for & all & \sigma \end{array} \end{array}$$

where $\dot{\varepsilon }$ is the strain rate, *A*_1_ is the material constant, *g*(*σ*) is a function of the flow stress, *Q* is the deformation activation energy (J/mol), *R* is the gas constant, 8.314 J/(mol·K), and *T* is the thermodynamic temperature (K), *A*, *n*, *n*_1_, *α* and *β* are the material-dependent thermal deformation constants, and *α* = *β*/*n*_1_ is satisfied.

In isothermal deformation, the true stress–strain relationship is affected by the deformation temperature and strain rate. The combined effect is usually expressed by the Zener–Hollomon parameter. The Equation (2) is the Z-parametric expression and the material constitutive equation for high-temperature flow stress [30].

(2) $$\left\{ \begin{array}{l} Z=\dot{\varepsilon }\;\exp\;\left[\;Q/\left(RT\right)\right]\\ \sigma =1/\alpha \ln\left\{{\left(Z/A\right)}^{1/n}+{\left[{\left(Z/A\right)}^{2/n}+1\right]}^{1/2}\right\} \end{array}\right.$$

**Figure 4:**
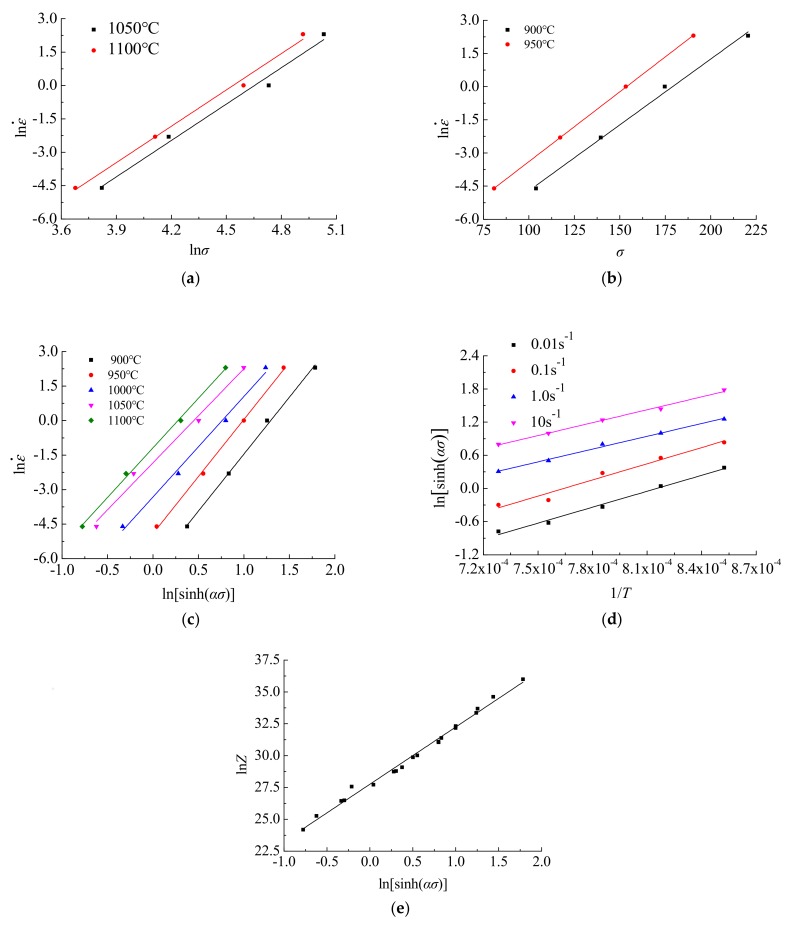
Correlation coefficient fitting: (**a**) In $\dot{\varepsilon }$-In*σ*; (**b**) In $\dot{\varepsilon }$-*σ*; (**c**) In $\dot{\varepsilon }$-In[sinh(*ασ*)]; (**d**) In[sinh(*ασ*)]-1/*T*; (**e**) In*Z*-In[sinh(*ασ*)].

The mathematical derivation of Equations (1) and (2) is performed. According to the experimental results of true stress-strain data and by linear regression of $\ln\dot{\varepsilon }-\ln\sigma$, $\ln\dot{\varepsilon }-\sigma$, $\ln\dot{\varepsilon }-\ln\left[\sinh\left(\alpha \sigma \right)\right]$, ln[sinh(ασ)]−1/T and lnZ−ln[sinh(ασ)], the relevant parameters in the flow stress model with *ε* = 0.3 are obtained as follows: *n*_1_ = 5.4488, *β* = 0.0163, *α* = 0.0113, *n* = 4.5368, *Q* = 328645.0625, *A* = 1.13251012. The linear fitting of correlation coefficients is shown in Figure 4.

### 2.3. Dynamic Recrystallization Model

#### 2.3.1. Kinetic Model

During thermal deformation, dynamic recrystallization begins when ε = ε_c_. Different materials have different ε_c_ at different deformation temperatures and strain rates. They are not only related to the deformation temperature and strain rate, but also related to the grain size and activation energy before deformation. At present, the dynamic recrystallization critical strain model is expressed by the Sellars model shown in Equation (3) [31,32,33].

(3) $$\left\{ \begin{array}{l} \varepsilon_{p}=a_{1}Z^{m_{1}}=a_{1}{\left[\dot{\varepsilon }\exp\left(\frac{Q_{1}}{RT}\right)\right]}^{m_{1}}\\ \varepsilon_{c}=a_{2}\varepsilon_{p} \end{array}\right.$$

where *ε_p_* is the peak strain (s^−1^), *ε_c_* is the critical strain (s^−1^), *Q*_1_ is dynamic recrystallization activation energy (J/mol), *Z* is the thermal deformation parameter of the thermal deformation process, *T* is the thermodynamic temperature (K), *a*_1_, *a*_2_, and *m*_1_ are the material-dependent thermal deformation constants.

The critical conditions for dynamic recrystallization to occur are shown in Equation (4).

(4) $$\varepsilon_{c}=\frac{\partial^{2}\ln\theta }{\partial \varepsilon^{2}}$$

where *θ* indicates the work hardening rate (MPa), *θ* = d*σ*/d*ε*.

The results of Mirzadeh and Najafizadeh [34] show that the critical conditions of dynamic recrystallization can be obtained by fitting the relationship between In*θ* and *ε*, shown as follows:

In*θ* = *A*_3_*ε*^3^ + *A*_2_*ε*^2^ + *A*_1_*ε* + *A*_0_ (5)

The dynamic recrystallization critical strain expression can be derived mathematically, as shown below:

(6) $$\varepsilon_{c}=\frac{\partial^{2}\ln\theta }{\partial \varepsilon^{2}}=-\frac{A_{2}}{3A_{3}}\;\left(0\leq \varepsilon \leq \varepsilon_{p}\right)$$

When ε = ε_c_, the microstructure change enters the dynamic recrystallization softening stage. As the strain increases, the flow stress gradually reaches its maximum value, and the work hardening rate become zero, shown as follows:

(7) $$\left\{ \begin{array}{l} \theta =\frac{\partial \sigma }{\partial \varepsilon }=0\\ \varepsilon_{p}=\varepsilon \end{array}\right.$$

**Figure 5:**
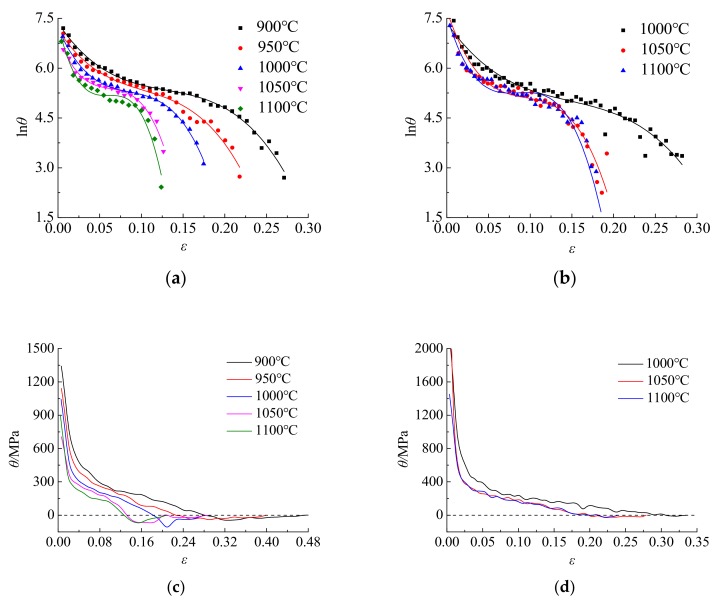
Critical strain and peak strain solution curve: (**a**) In*θ*-ε ($\dot{\varepsilon }$ = 0.01 s^−1^); (**b**) In*θ*-ε ($\dot{\varepsilon }$ = 0.1 s^−1^); (**c**) In*θ*-ε ($\dot{\varepsilon }$ = 0.01 s^−1^); (**d**) In*θ*-ε ($\dot{\varepsilon }$ = 0.1 s^−1^).

According to the experimental true stress-strain data, ε_c_ and ε_p_ are obtained by the cubic polynomial nonlinear fitting curves of In*θ*-ε and *θ*-ε. The dynamic recrystallization critical strain and peak strain value are fitted to the fitting curve, as shown in Figure 5. The values of the critical strain ε_c_ and the peak strain ε_p_ are shown in Table 3.

The natural logarithm of Equation (3) is taken and Equation (8) is obtained as follows:

(8) $$\ln\varepsilon_{p}=\ln a_{1}+m_{1}\ln\dot{\varepsilon }+m_{1}\frac{Q_{1}}{RT}$$

**Figure 6:**
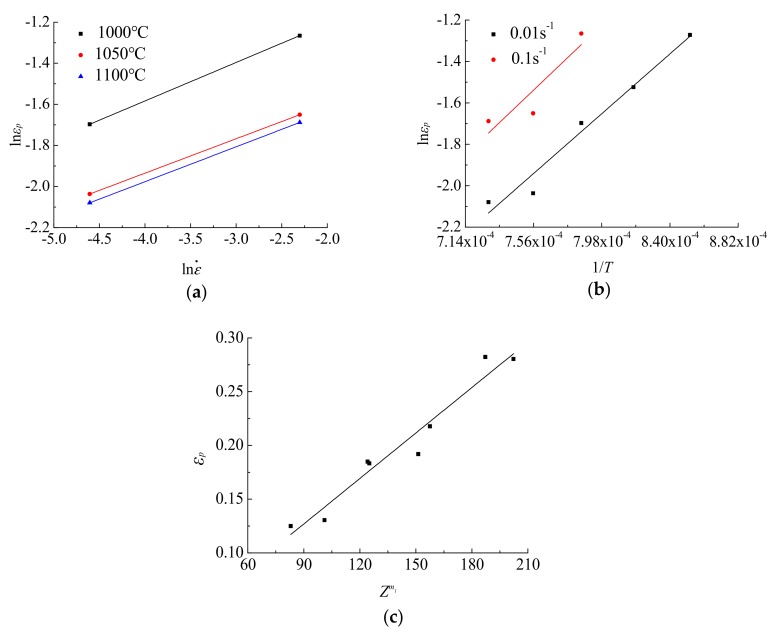
Material constant linear regression solution: (**a**) Inε_p_-In $\dot{\varepsilon }$; (**b**) Inε_p_-1/*T*; (**c**) ε_p_-*Z*^*m*1^.

When the deformation temperature $\sigma_{s}$ and the strain rate $\dot{\varepsilon }$ are constant values, respectively, linear regression is used to obtain *m*_1_, *m*_1_*Q*_1_/*R*, *a*_1_ and *a*_2_, as shown in Figure 6.

Solved by using linear regression, the parameters of the Sellars critical strain model are as follows: *m*_1_ = 0.1749, *Q*_1_ = 340963.2594, *a*_1_ = 0.0014 and *a*_2_ = 0.4969.

#### 2.3.2. Kinematic Model

The dynamic recrystallization Kinematics model is generally expressed in the Avrami equation [35,36], as shown below:

(9) $$\left\{ \begin{array}{cc} X_{Drex}=0 & \left(0\leq \varepsilon \leq \varepsilon_{c}\right)\\ X_{Drex}=1-\exp\left[-\beta_{d}{\left(\frac{\varepsilon -\varepsilon_{c}}{\varepsilon_{0.5}}\right)}^{k}\right] & \left(\varepsilon \geq \varepsilon_{c}\right)\\ \varepsilon_{0.5}=a_{3}{\dot{\varepsilon }}^{m_{2}}\exp\left(\frac{Q_{2}}{RT}\right) & \end{array}\right.$$

where *X_Drex_* is the dynamic recrystallization volume percentage, *ε*_0.5_ is the strain value when the dynamic recrystallization volume percentage is 50%, *Q*_2_ is the activation energy (J/mol) when the dynamic recrystallization volume percentage is 50%, *β_d_*, *k*, *α*_3_ and *m*_2_ are the thermal deformation constants associated with the material.

Sellars proposed a method for obtaining the dynamic recrystallization volume percentage by a mathematical model, the expression of which is shown in Equation (10) [37].

(10) $$X_{Drex}=\frac{\sigma_{WH}-\sigma }{\sigma_{s}-\sigma_{ss}}\;\left(\varepsilon \geq \varepsilon_{c}\right)$$

where *σ_WH_*, *σ* are the dynamic stress curve and the dynamic recrystallization curve flow stresses (MPa), respectively, *σ_s_* is the dynamic recovery curve steady state stress (MPa), *σ_ss_* is the dynamic recrystallization curve steady state stress (MPa). Among these parameters, *σ_ss_* and *σ* can be directly obtained from the dynamic recrystallization curve, while *σ_WH_* and *σ_s_* can be directly obtained from the dynamic recovery curve.

The dynamic response curve derived using the method proposed by Sellars is shown in Equation (11).

(11) $$\frac{\sigma_{WH}-\sigma_{0.2}}{\sigma_{s}-\sigma_{0.2}}={\left[1-\exp\left(-b\varepsilon \right)\right]}^{c}$$

where *σ*_0.2_ is the flow stress when the compressive deformation is 0.2%, *b* and *c* are constants related to material properties. Equation (12) can be derived mathematically from Equation (11).

(12) $$\sigma^{\prime }=a_{4}{\left[1-\exp\left(-b\varepsilon \right)\right]}^{c}$$

**Figure 7:**
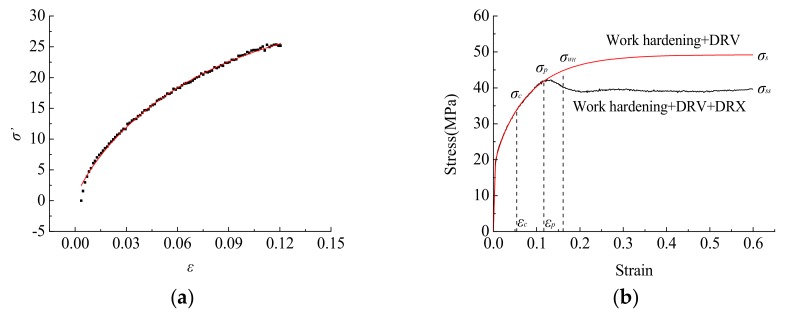
Dynamic recovery and dynamic recrystallization curve. (**a**) Dynamic recovery curve; (**b**) Dynamic recrystallization curve.

According to the experimental true stress–strain data, the nonlinear data fitting of Equation (12) is obtained, and it is obtained that: *a*_4_ = 48.6483, *b* = 6.4622, *c* = 0.4164. Figure 7 shows the dynamic recovery and dynamic recrystallization curves.

By combining Equation (10) with Equation (12), the dynamic recrystallization volume fraction can be expressed as follows:

(13) $$X_{Drex}=\frac{\sigma_{WH}-\sigma }{\sigma_{s}-\sigma_{ss}}=\frac{a{\left[1-\exp\left(-b\varepsilon \right)\right]}^{c}+\sigma_{0.2}-\sigma }{\sigma_{s}-\sigma_{ss}}$$

According to Equation (13), the strain value ε_0.5_ is obtained when the dynamic recrystallization volume percentage is 50%. And the strain values, when the dynamic recrystallization volume percentage is 50% under different test conditions, are shown in Table 4.

When *ε* ≥ *ε_c_*, the natural logarithm of Equation (11) is taken and Equations (14) and (15) are obtained:

(14) $$\ln\left[-\ln\left(1-X_{Drex}\right)\right]=\ln\beta_{d}+k\ln\left[\left(\varepsilon -\varepsilon_{c}\right)/\varepsilon_{0.5}\right]\;\left(\varepsilon \geq \varepsilon_{c}\right)$$

(15) $$\ln\varepsilon_{0.5}=\ln a_{3}+m_{2}\ln\dot{\varepsilon }+Q_{2}/\left(RT\right)$$

By the linear regression for ln[−ln(1−X_Drex_)]-ln[(ε−ε_c_)/ε_0.5_], lnε_0.5_-ln $\dot{\varepsilon }$, lnε_0.5_-1/*T*, the dynamic recrystallization kinematic model parameters are obtained as follows: *k* = 4.3829, *β* = 3.2731, *Q*_2_ = 49840.7094, *m*_2_ = 0.1520, *a*_3_ = 0.0042.

#### 2.3.3. Dynamic Recrystallization Grain Size Model

The Gleeble-3800 compression test piece is cut in the radial direction, and the dynamic recrystallized grain size is obtained by etching the sample with a supersaturated picric acid aqueous solution and a small amount of an alkaline reagent, as shown in Table 5.

The deformation temperature and strain rate affect the dynamic recrystallized grain size, and Equation (16) is used to describe the dynamic recrystallized grain size change.

(16) $$d_{DRX}=a_{5}{\dot{\varepsilon }}^{m_{3}}\exp[Q_{3}/\left(RT\right)]$$

where *d_DRX_* is the dynamic recrystallization grain size (μm), *a*_5_ and *m*_3_ are the material correlation coefficients, *Q*_3_ is the grain growth activation energy.

The logarithm of the two sides of Equation (16) is taken and Equation (17) is obtained.

(17) $$\ln d_{DRX}=\ln a_{5}+m_{3}\ln\dot{\varepsilon }+Q_{3}/\left(RT\right)$$

**Figure 8:**
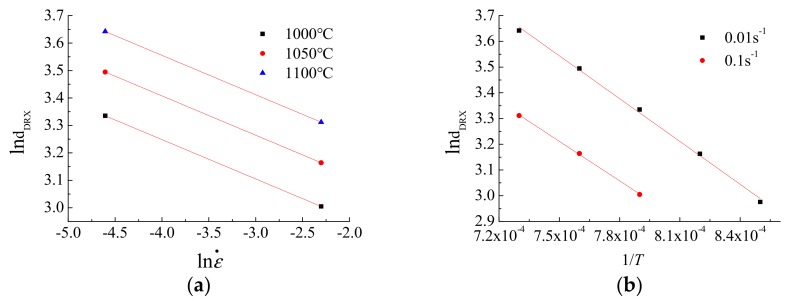
Linear regression results of dynamic recrystallization equation: (**a**) lnε_p_-ln $\dot{\varepsilon }$; (**b**) lnε_p_-1/T.

The linear regression of ln $\dot{\varepsilon }$ -ln*d_DRX_* and 1/*T*-ln*d_DRX_* is performed on the two relations, and the relationship is obtained as shown in Figure 8. Finally, the parameters are calculated as: *m*_3_ = −0.1434, *Q*_3_ = −44330.25. Substituting *m*_3_ and *Q*_3_ into Equation (17), and result obtained was *a*_5_ = 981.7866.

## 3. Finite Element (FE) Analysis of Shear Connection Process

**Figure 9:**
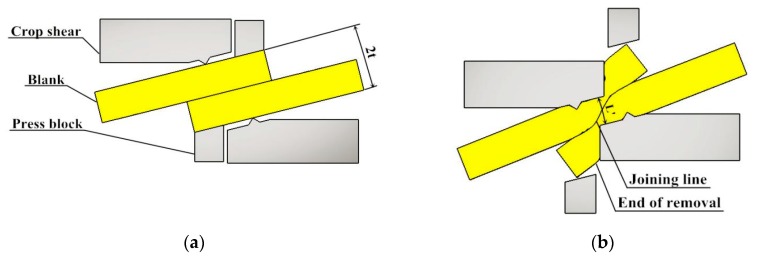
Process of cut connection: (**a**) before cutting the connection; (**b**) after cutting the connection.

Development of Deform-3D (Version: Deform6.01) Software Developed by SFTC Company in USA (Santa Fe, NM, USA), and the established Arrhenius constitutive equation and dynamic recrystallization model are placed into the user subroutine. The core codes of user subroutines are written in FORTRAN language and stored in Deform-3D V6_0 User Routine def_usr. In this paper, the constructed constitutive model and dynamic recrystallization model are embedded in the DEF_SIM folder of Deform. The grain size and volume percentage of dynamic recrystallization can be obtained by simulating the shear connection process. The effects of different shear connection conditions on the structure evolution, especially the dynamic recrystallization process, were discussed, and the appropriate shear connection parameters were found. The simulated shear connection process is shown in Figure 9. The upper crop shear is descending, and the shear edge contacts the blank to plastically deform the blank. Then the edge bites into the blank, and the blank undergoes severe deformation under the action of the cutting edge to achieve a quick shear connection. The degree of shear connection is characterized by the pressure amount L, the expression of which is shown in Equation (18).

(18) $$L=\frac{2t-L^{\prime }}{t}\times 100\%$$

where *t* is the thickness of a single sheet, *L*′ is the thickness of the sheet after shearing.

**Figure 10:**
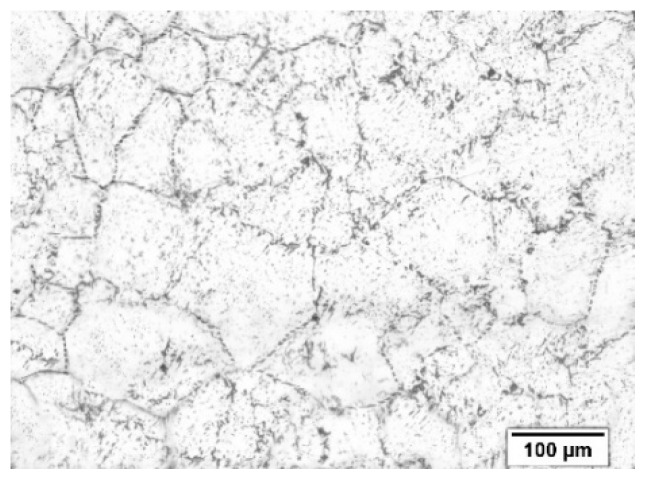
Q345 steel prior austenite structure.

The prior austenite grain size of about 120 μm is obtained by using a supersaturated picric acid aqueous solution plus a small amount of an alkaline reagent, as shown in Figure 10. Based on Deform-3D with rigid-plastic model, the FE model of the shear connection is established to explore the dynamic recrystallization degree and temperature change during the shear connection. The detailed parameters of the FE model are listed in Table 6. Element type, tetrahedron; element number, 20,000; node number, 4085; and min mesh size, 1.66 mm.

During the shearing connection process, the blank first contact the upper and lower shear edges, and slight plastic deformation occurs. As the upper shear descends, the pressure amount increases, and the blank undergoes plastic deformation by the action of the shear edge. With the increase of the pressure amount, the plastic deformation gradually extends to the overlapping surfaces of the two blanks along the line connecting the two cutting edges. The plastic deformation area of the upper and lower blanks merges, and the joint surface is gradually formed. As the shear connection continues, the dynamic recrystallization behavior of the plastic deformation area continues to expand.

**Figure 11:**
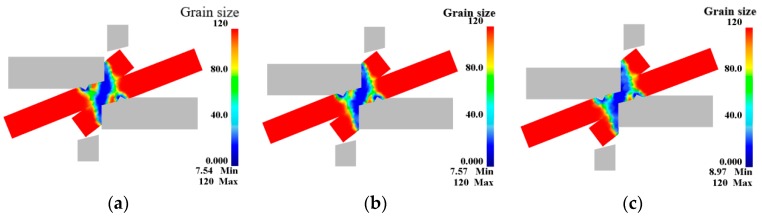
Dynamic recrystallized grain size: (**a**) L = 100%; (**b**) L = 125%; (**c**) L = 150%.

**Figure 12:**
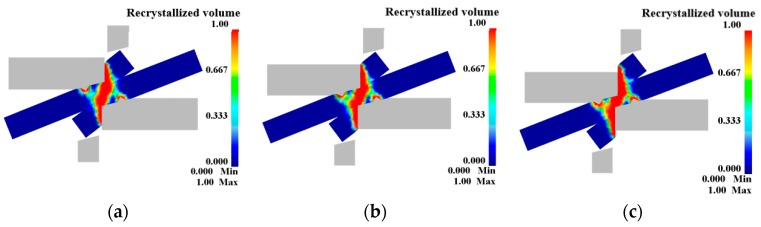
Dynamic recrystallization volume percentage: (**a**) L = 100%; (**b**) L = 125%; (**c**) L = 150%.

Figure 11 and Figure 12 show the simulation results. The blank is undeformed at both ends and no dynamic recrystallization occurs (the dynamic recrystallization volume percentage is 0%). The center of the shearing action (blue color area in Figure 11) is a large deformation area, and significant dynamic recrystallization occurs in this area with the grains refined. The reason for this phenomenon is that the shearing edge undergoes severe plastic deformation and a large amount of slip and dislocation is produced under the friction at the interface of the blank, which induces a large amount of dynamic recrystallization. The remaining area (light green area in Figure 11) is a small deformation area. Only upsetting-like deformation occurs in this region, and dynamic recrystallization is insufficient.

**Figure 13:**
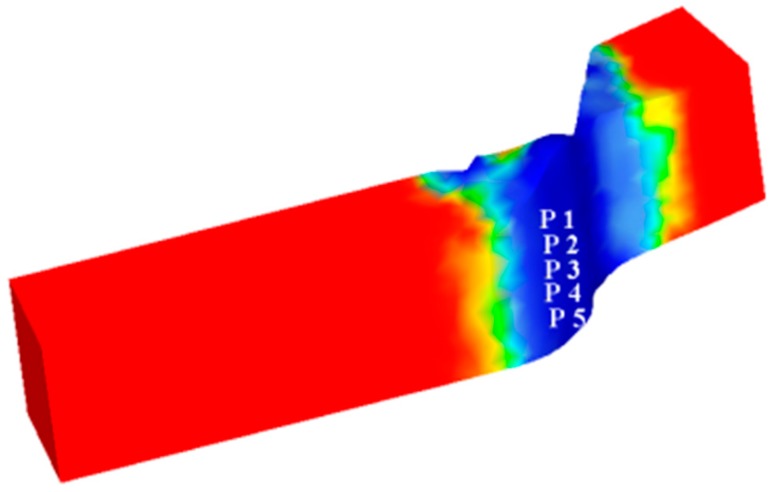
Tracking point location.

The point tracking method is used and five tracking points are taken near the blank connection surface in the direction perpendicular to the width of the blank, as shown in Figure 13. The changes of dynamic recrystallized grain size and volume percentage during the shear connection of the five tracking points are acquired.

**Figure 14:**
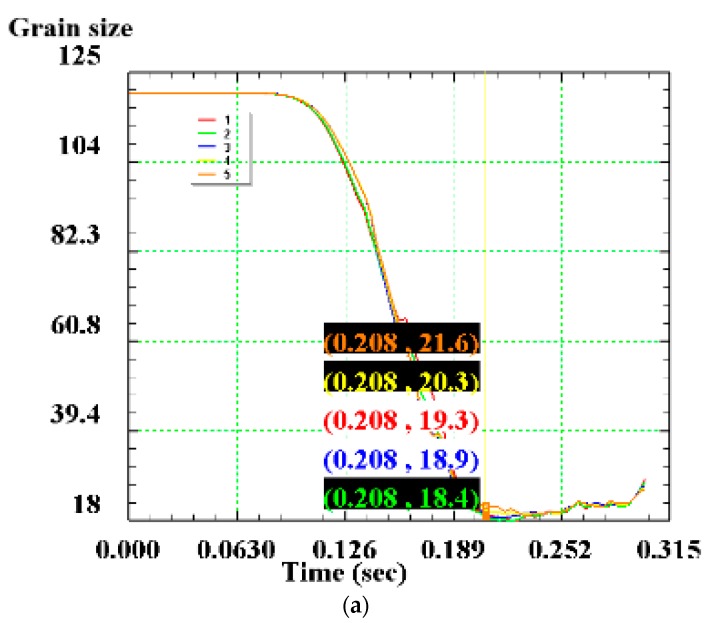

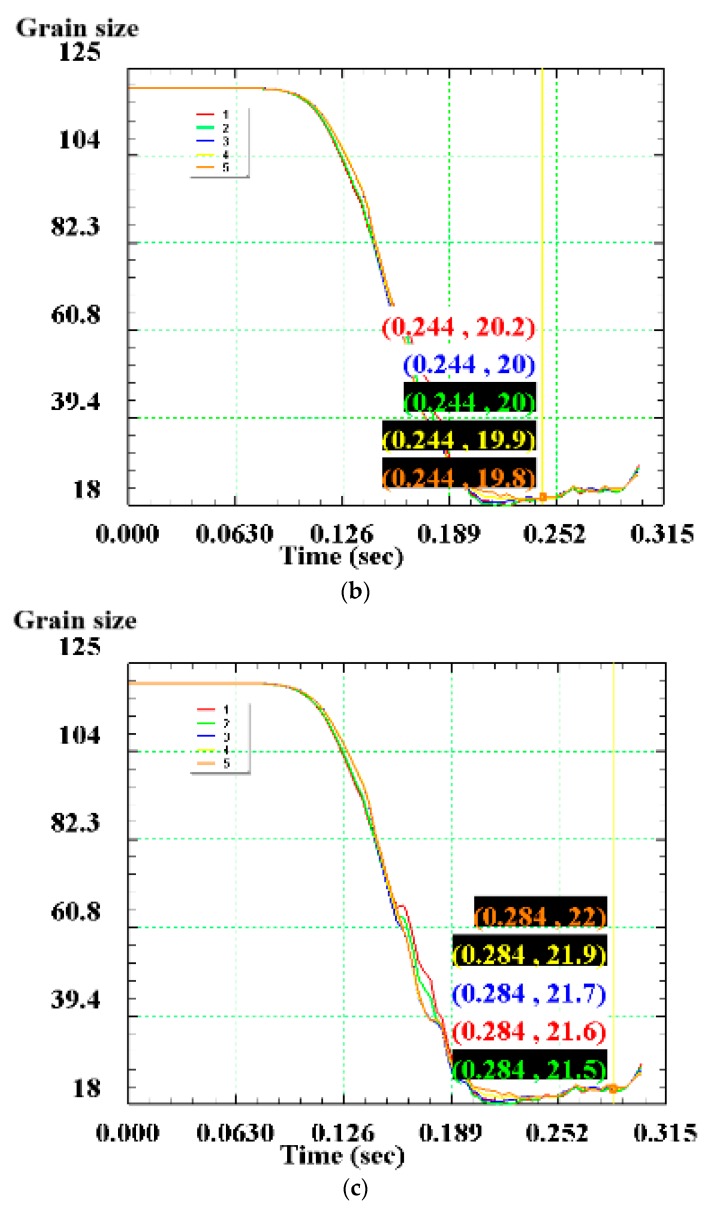
Dynamic recrystallized grain size at each tracking point: (**a**) L = 100%; (**b**) L = 125%; (**c**) L = 150%.

**Figure 15:**
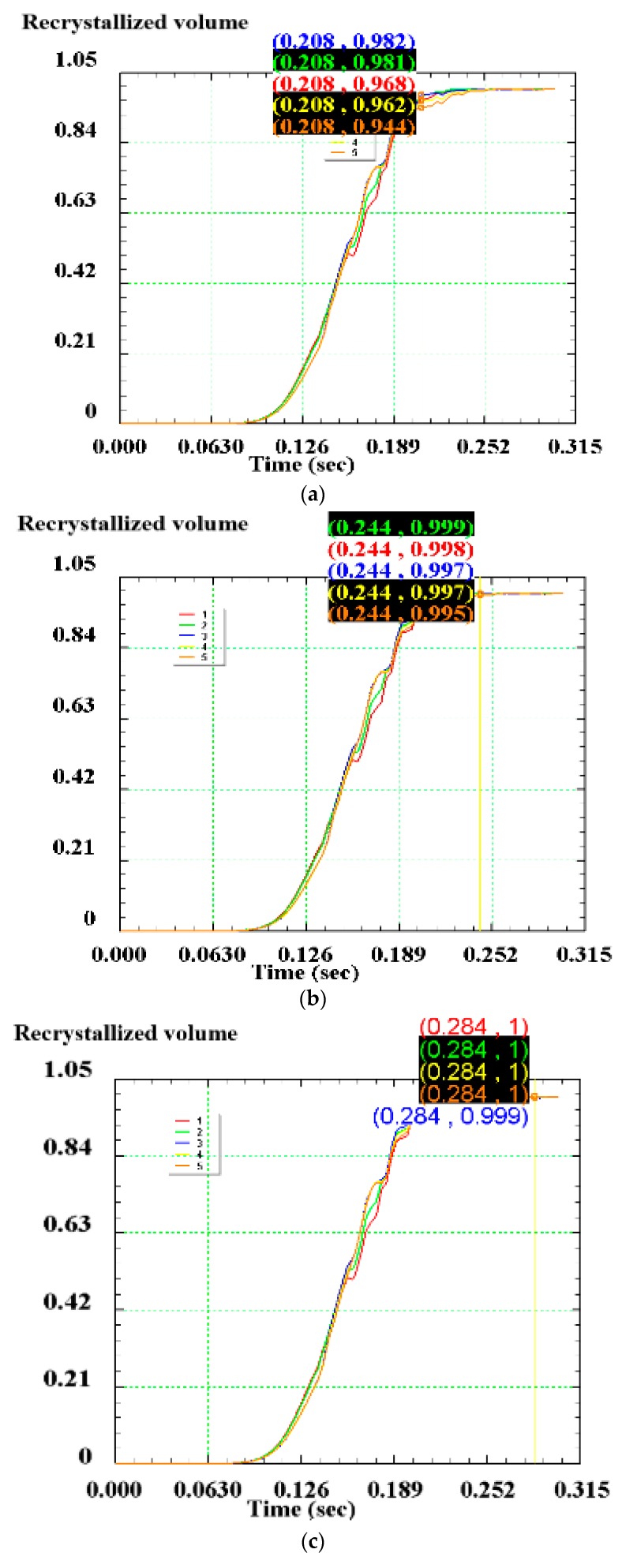
Dynamic recrystallization volume percentage of each tracking point: (**a**) L = 100%; (**b**) L = 125%; (**c**) L = 150%.

**Figure 16:**
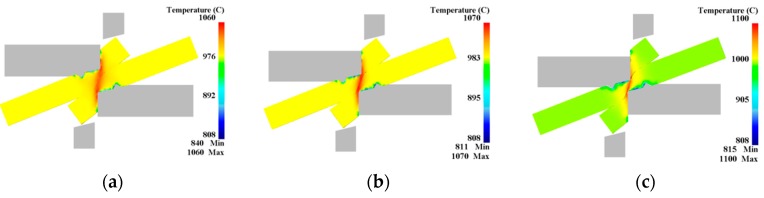
Temperature changes during the shear connection process: (**a**) L = 100%; (**b**) L = 125%; (**c**) L = 150%.

The relationship between the dynamic recrystallized grain size, volume percentage and the pressure amount in the large deformation area of the blank is shown in Figure 14 and Figure 15. The change of grain size with the shear pressure amount is reflected: the grain size gradually decreases with the increase of the pressure amount, but after the pressure amount reaches 100%, the grain size increases slightly. The reason for this might be that part of the distortion energy inside the grain is converted into heat (see Figure 16), and the remaining distortion energy is insufficient to maintain the formation of new fine grains of recrystallization.

The dynamic recrystallization volume fraction changes with the pressure amount: as the pressure amount increases, the dynamic recrystallization volume percentage also increases, and the curve of the dynamic recrystallization volume percentage with the pressure amount also gradually converges to 100%. This is because as the pressure amount increases, the dynamic recrystallized grains continue to grow, and new recrystallized grains are continuously formed, eventually causing the matrix structure to be stable in an equiaxed state. At this time, the dynamic recrystallization volume percentage is 100%. When L = 100%, the volume percentage reaches 96.74%, the grain size is refined to 19.7 μm; when L = 125%, the volume percentage reaches 99.72%, the grain size is 19.98 μm; when L = 150%, the volume percentage reached 100%, and complete dynamic recrystallization is achieved with a grain size of 21.74 μm. The specific values are shown in Table 7.

## 4. Shear Connection Experiment

**Figure 17:**
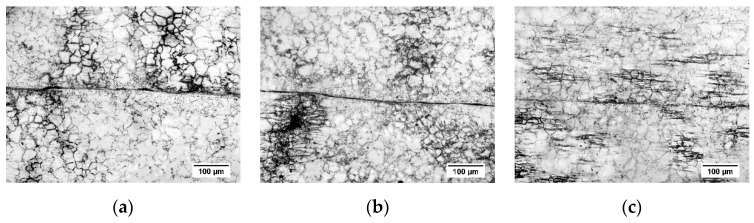
Microscopic morphology at different pressure amounts: (**a**) L = 100%; (**b**) L = 125%; (**c**) L = 150%.

In this experiment, a 315 t hydraulic press was used and the shear connection experiment is carried out under the physical simulation experimental conditions. The microscopic state under different pressure amounts is analyzed to verify the accuracy of the model. Figure 17 shows the microstructure of the large deformation area when the pressure amount is 100%, 125% and 150%, respectively. With the increase of the pressure amount, the grain size is refined remarkably, which is consistent with the simulation trend. This is because of the early stage of the shear connection process, the plastic deformation process at high temperature causes dynamic recrystallization of grains, severe shear and friction of overlapped sheets, and energy input to the sheets, which further increases the energy. With the shear deformation process, oxide scales and other impurities continue to break up, forming fine particles, which provides a nucleation site for dynamic recrystallization. Therefore, the grain size is refined continuously during shear connection. After the pressure amount reaches 100%, the grain size increases slightly. The reason for this might be that part of the distortion energy inside the grain is converted into heat (see Figure 16), and the remaining distortion energy is insufficient to maintain the formation of new fine grains of recrystallization. At this time, driven by temperature, atoms diffuse and grains grow relatively.

The dynamic recrystallization grain size is measured by using composite grid method according to the ASTM standard. The results of the shear connection experiment and numerical simulation of austenite grain size are shown in Table 8. When the pressure amount is 100%, 125%, 150%, respectively, the grain size is about 19.16 μm, 20.25 μm, 22.21 μm, respectively. From these results, it is found that the grain size obtained by the experiment and the simulation are basically the same, and the average error is less than 3%, indicating the constitutive and dynamic recrystallization model established in this paper has high accuracy.

## 5. Conclusions

(1) In this paper, the true stress–strain curve of the material is obtained by the thermal compression simulation test, and the constitutive equation for high-temperature flow stress and the dynamic recrystallization model are constructed.

The high-temperature flow stress constitutive model is shown as follows:

(19) $$\left\{ \begin{array}{l} \sigma =1/0.0113\ln\left\{\;{\left(Z/1.1325\times 10^{12}\right)}^{1/4.5368}+{\left[{\left(Z/1.1325\times 10^{12}\right)}^{2/4.5368}+1\right]}^{1/2}\;\right\}\\ Z=\dot{\varepsilon }\;\exp\left[\;328645.0625/\left(RT\right)\right] \end{array}\right.$$

The dynamic recrystallization kinetics model is shown as follows:

(20) $$\left\{ \begin{array}{l} \varepsilon_{p}=0.0014Z^{0.1749}=0.0014{\left[\dot{\varepsilon }\exp\left(\frac{340963.2594}{RT}\right)\right]}^{0.1749}\\ \varepsilon_{c}=0.4969\varepsilon_{p} \end{array}\right.$$

The dynamic recrystallization kinematic model is shown as follows:

(21) $$\left\{ \begin{array}{cc} X_{Drex}=0 & \left(0\leq \varepsilon \leq \varepsilon_{c}\right)\\ X_{Drex}=1-\exp\left[-2.4630{\left(\frac{\varepsilon -\varepsilon_{c}}{\varepsilon_{0.5}}\right)}^{4.1332}\right] & \left(\varepsilon \geq \varepsilon_{c}\right)\\ \varepsilon_{0.5}=0.0042{\dot{\varepsilon }}^{0.1520}\exp\left(\frac{49840.7094}{RT}\right) & \end{array}\right.$$

The dynamic recrystallization grain size model is shown as follows:

(22) $$d_{DRX}=981.7866{\dot{\varepsilon }}^{-0.1434}\exp\left(\frac{-44330.25}{RT}\right)$$

(2) The finite element simulation results show that the shear connection process is divided into three areas, namely the large deformation area, small deformation area and undeformed area. The dynamic recrystallization volume percentage increases with the increase of the pressure amount. When the pressure amount is 100%, 125% and 150%, respectively, the grain size is 19.7 μm, 19.98 μm, 21.74 μm, respectively, and the volume percentage is 96.74%, 99.72%, 100%, respectively.

(3) The experiment shows that when the pressure amount is 100%, 125% and 150%, respectively, the grain size is 19.2 μm, 20.3 μm and 22.2 μm, respectively.

By comparison, it is found that the experimental results are consistent with those of the numerical simulation, and the average error is less than 3%.

## Figures and Tables

**Table 1:** Chemical component of Q345.

Chemical Element	C	Mn	S	Cr	Ni	Si	Fe
Element Content (wt %)	0.18	0.42	0.0098	0.006	0.013	0.147	Bal.

**Table 2:** Main parameters of Gleeble-3800 thermal simulator.

Main Parameters	Value
Maximum Heating Rate	10000 °C/s
Maximum Quenching Rate	1000 °C/s
Maximum Stroke	100 mm
Maximum Stroke Rate	2000 mm/s
Maximum Compression Tonnage	20 t
Maximum Tensile Tonnage	10 t
Maximum Sample Size	Φ20 mm
Temperature Range	room temperature~1700 °C
Resolving Power	1 °C

**Table 3:** Critical strain and peak strain.

$\dot{\varepsilon }$ (s^−1^)	T (°C)	ε_c_	ε_p_
0.01	900	0.1302	0.2804
0.01	950	0.1030	0.2178
0.01	1000	0.0861	0.1832
0.01	1050	0.0623	0.1305
0.01	1100	0.0608	0.1250
0.10	1000	0.1475	0.2822
0.10	1050	0.0945	0.1919
0.10	1100	0.0893	0.1848

**Table 4:** ε_0.5_ values under different test conditions.

$\dot{\varepsilon }$ (s^−1^)	T (°C)	ε_0.5_
0.01	900	0.3352
0.01	950	0.2945
0.01	1000	0.2364
0.01	1050	0.1809
0.01	1100	0.1653
0.10	1000	0.3419
0.10	1050	0.2284
0.10	1100	0.2455

**Table 5:** Dynamic recrystallized grain size under various deformation conditions.

$\dot{\varepsilon }$ (s^−1^)	T (°C)	d_DRX_ (μm)
0.01	900	19.6
0.01	950	23.6
0.01	1000	28.1
0.01	1050	32.9
0.01	1100	38.2
0.10	1000	20.2
0.10	1050	23.7
0.10	1100	27.4

**Table 6:** Detailed parameters of finite element (FE) model.

Parameter	Blank Temperature	Blank Size	Shear Speed	λ_1_	λ_2_
Value	1000 °C	120 × 29 × 21.75 mm	150 mm/s	0.45 N/(s·mm·C)	5 N/(s·mm·C)

λ_1_ is the friction coefficient of deformation. λ_2_ is the heat transfer coefficient between the blank and the die.

**Table 7:** Tracking point dynamic recrystallization grain size and volume percentage average.

Shear Pressure Amount (%)	Average Grain Size d_0_ (μm)	Average Volume Percentage X_Drx_ (%)
100	19.7	96.74
125	19.98	99.72
150	21.74	100

**Table 8:** Grain size during shear connection process.

Shear Pressure Amount (%)	Experimental Average Grain Size d (μm)	Simulated Grain Average Size d0 (μm)	Average Error (%)
100	19.2	19.7	2.6
125	20.3	19.98	1.1
150	22.2	21.74	2.1
